# Supplementary material for: Enhancement Effects and Mechanism Studies of Two Bismuth-Based Materials Assisted by DMSO and Glycerol in GC-Rich PCR
Source: Molecules. 2023 Jun 2;28(11):4515. doi: 10.3390/molecules28114515 (PMC10254864; doi:10.3390/molecules28114515)
Supplement: Supplementary file 1 [file molecules-28-04515-s001.zip › molecules-2345293-supplementary.pdf]

Supplementary materials

Enhancement Effects and Mechanism Studies of Two Bismuth-Based Materials Assisted by DMSO and Glycerol in GC-Rich PCR

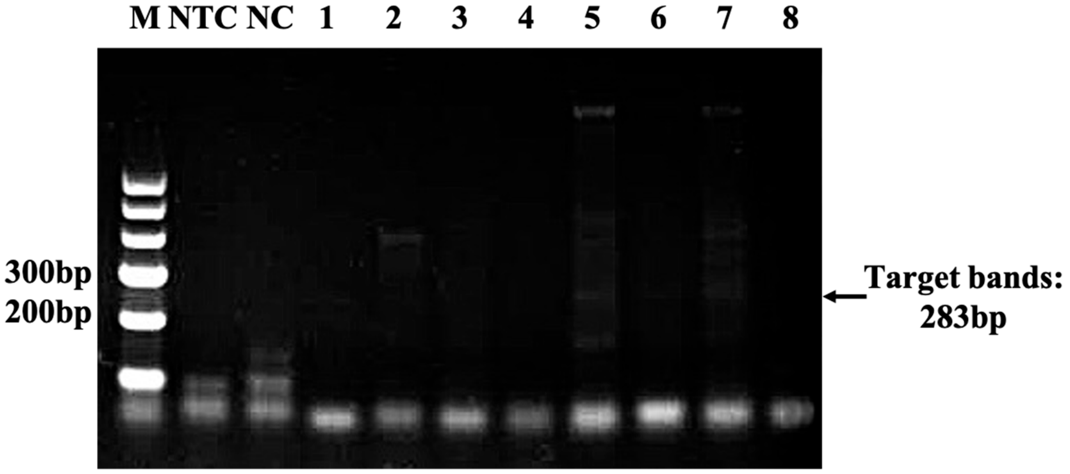

Figure S1. PCR amplification of Ex Taq DNA polymerase-mediated GNAS1 promoter was optimized using different ratios of enzymes, DMSO, glycerol with 2mM Mg^{2+}. Lane M: 100bp DNA Ladder (DL2000 Plus, Vazyme; DL1001，Generay）； lane NTC: no template control; Lane NC: negative control with template; Lanes 1-8: Optimal amplification of combinations of enzymes, DMSO, and glycerols in different proportions, as detailed in Table 2.

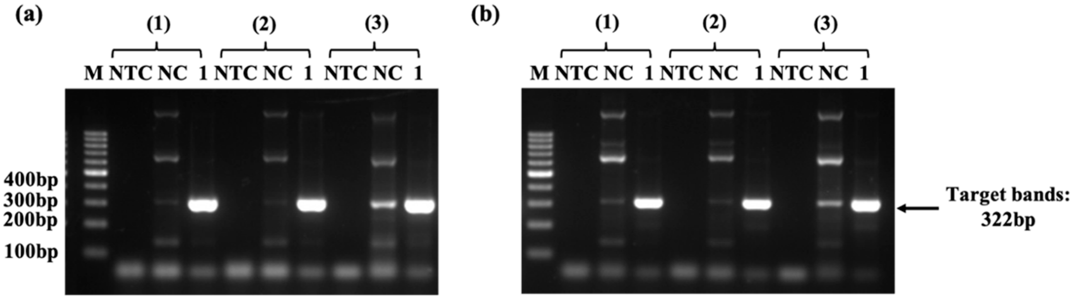

Figure S2. Optimization of the PCR amplification system of the Ex Taq DNA polymerase-mediated APOE gene. Optimized using 3%DMSO and 5%glycerol in two systems containing different concentrations of primers and polymerases, g-DNA extracted from different cells and g-DNA obtained were purchased, respectively, (1) RD cells, (2) U87-MG cells, and (3) purchased from bio cwbiotech Co., Ltd. (a) 0.4μM primer, 2.0U polymerase; (b) 0.2μM primer, 1.25U polymerase. Lane M: 100bp DNA Ladder (DL1002, Generay); lane NTC: no template control; Lane NC: negative control with template; Lane 1: 3%DMSO + 5%glycerol.

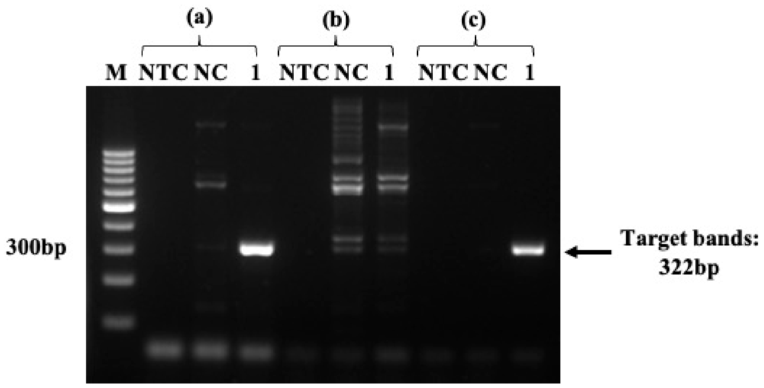

Figure S3. Optimization of Ex Taq DNA polymerase-mediated PCR amplification procedure for APOE genes. Optimized using 3%DMSO and 5%glycerol by three different PCR procedures: (a) annealed at 60°C for 45s for 35 cycles, (b) annealed at 60°C for 30s for 35 cycles, and (c) annealed at 60 °C and extended 20s for 30 cycles. Lane M: 100bp DNA Ladder (DL1002, Generay); lane NTC: no template control; Lane NC: negative control with template; Lane 1: 3%DMSO + 5%glycerol.

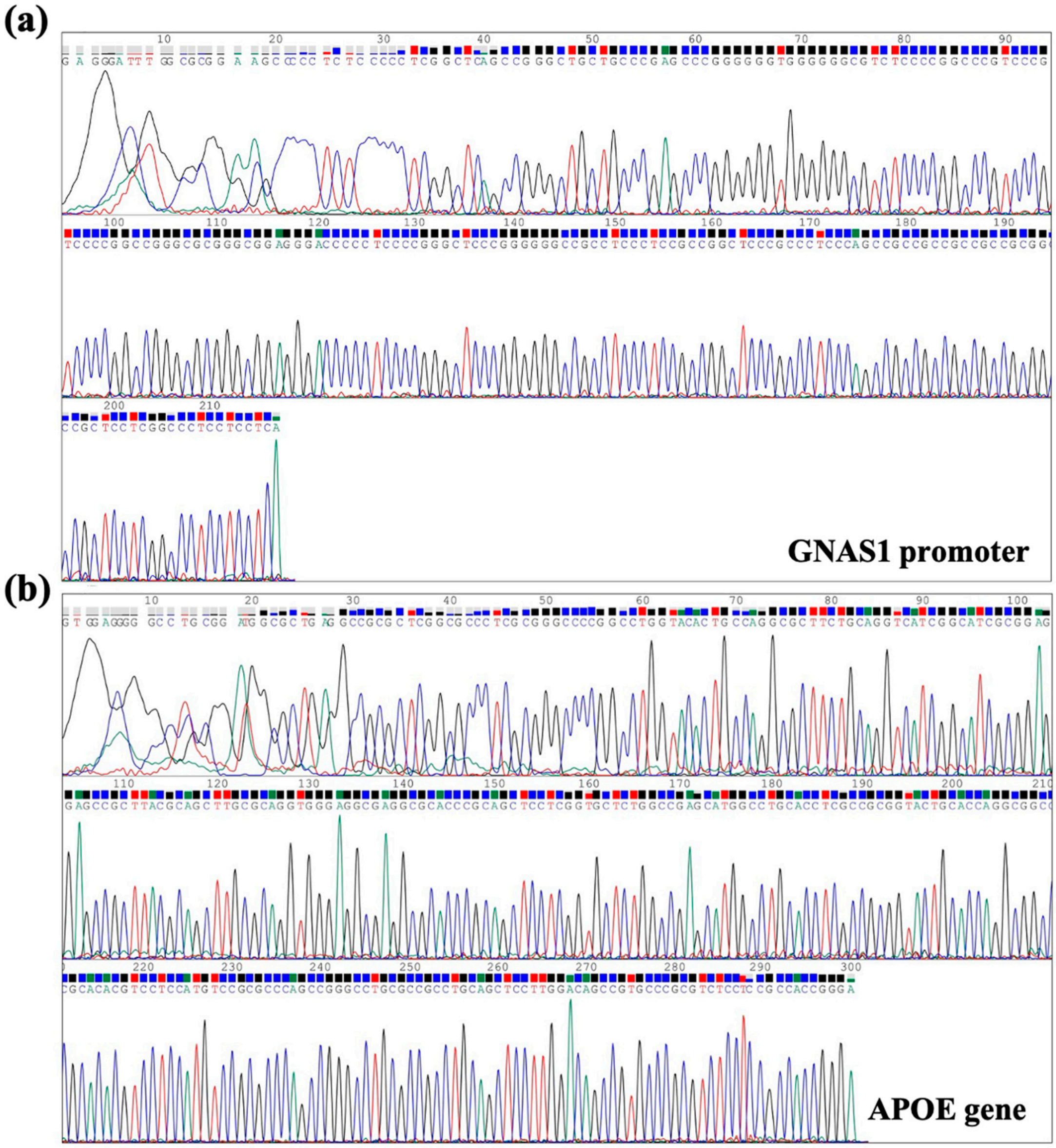

Figure S4. Sequencing map of the amplification product for (a) GNAS1 promoter and (b) APOE gene.

Table S1. BLAST comparison of sequencing results.

Gene name	Approximate species	Scientific name	Approximate rate
GNAS1	Homo sapiens GNAS complex locus (GNAS)	Homo sapiens	98.58%
APOE	Homo sapiens apolipoprotein E (APOE)	Homo sapiens	100.00%

Table S2. Comparison of the operating environment of different types of polymerases.

Polymerase type	Storage buffer							10× PCR buffer		
	Tris-HCl	KCl	EDTA	DTT	Tween 20	NP-40	Glycerol	Tris-HCl	KCl	NP-40
Takara Ex Taq	20 mM (pH 8.0)	100 mM	0.1 mM	1 mM	0.5%	0.5%	50.0%	100 mM (pH 8.9)	500 mM	-
Vazyme Taq	-	-	-	-	-	-	-	-	-	-
BBI Taq	20 mM (pH 8.0)	100 mM	0.1 mM	1 mM	0.5%	0.5%	50.0%	100 mM (pH8.8)	500 mM	0.8% (v/v)
NEB Taq	-	-	-	-	-	-	-	100 mM (pH 8.3)	500 mM	-
Takara rTaq	20 mM (pH 8.0)	100 mM	0.1 mM	1 mM	0.5%	0.5%	50.0%	100 mM (pH 8.9)	500 mM	-
Genstar Taq	-	-	-	-	-	-	-	100 mM (pH 8.3)	500 mM	-
Toyobo rTaq	20 mM (pH 8.0)	100 mM	0.1 mM	1 mM	0.5%	0.5%	50.0%	-	-	-

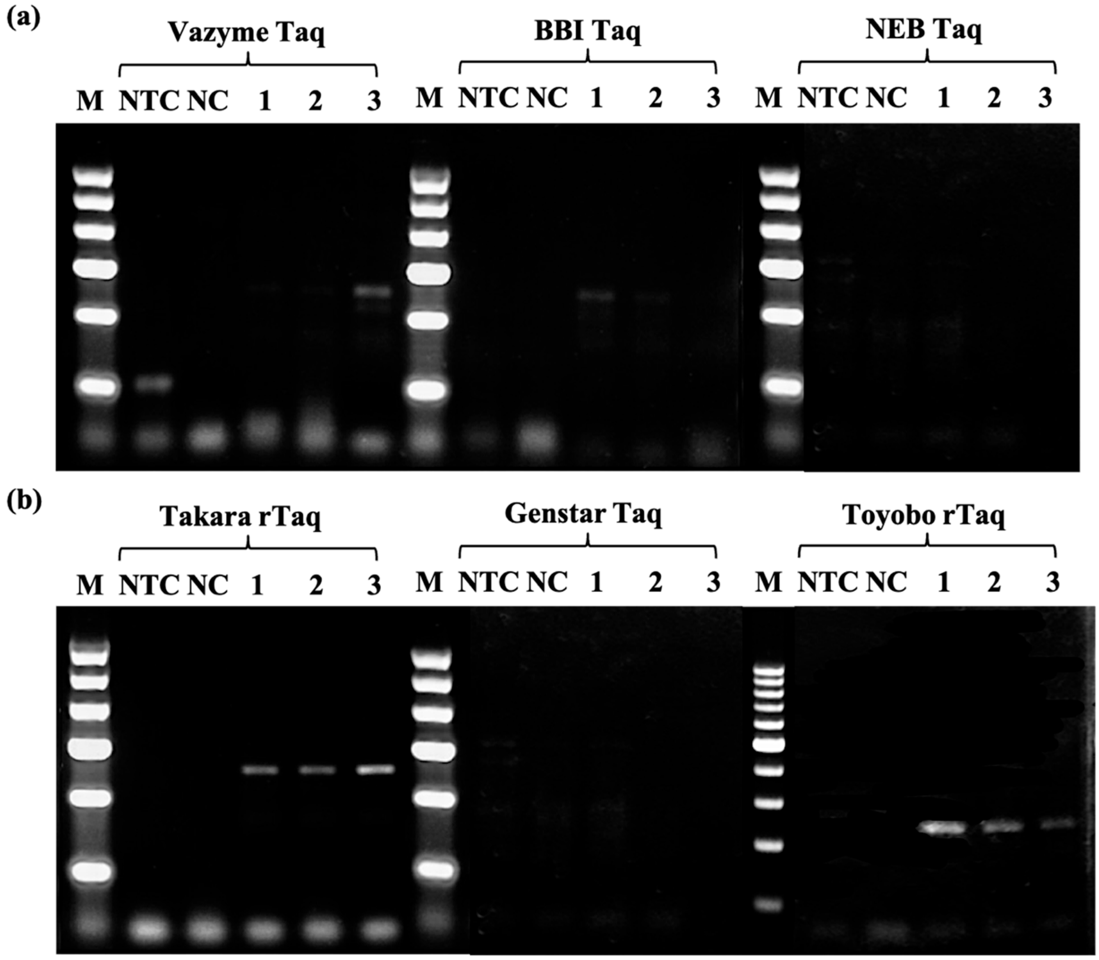

Figure S5. Applicability of using different brands of Taq DNA polymerase in PCR systems for bismuth compound-enhanced amplification of GNAS1 promoters. (a) Vazyme Taq, BBI Taq, NEB Taq and (b) Takara rTaq, Genstar Taq, Toyobo rTaq DNA polymerases amplified under the action of ammonium bismuth citrate or bismuth subcarbonate, respectively. Lanes 1-3: 0nM material (3%DMSO and 5%glycerol), 0.22nM ammonium bismuth citrate and 0.1mM bismuth subcarbonate. Lane M: 100bp DNA Ladder (DL1001 and DL1002, Generay); Swimlane NTC: no template control; Lane NC: negative control with template.

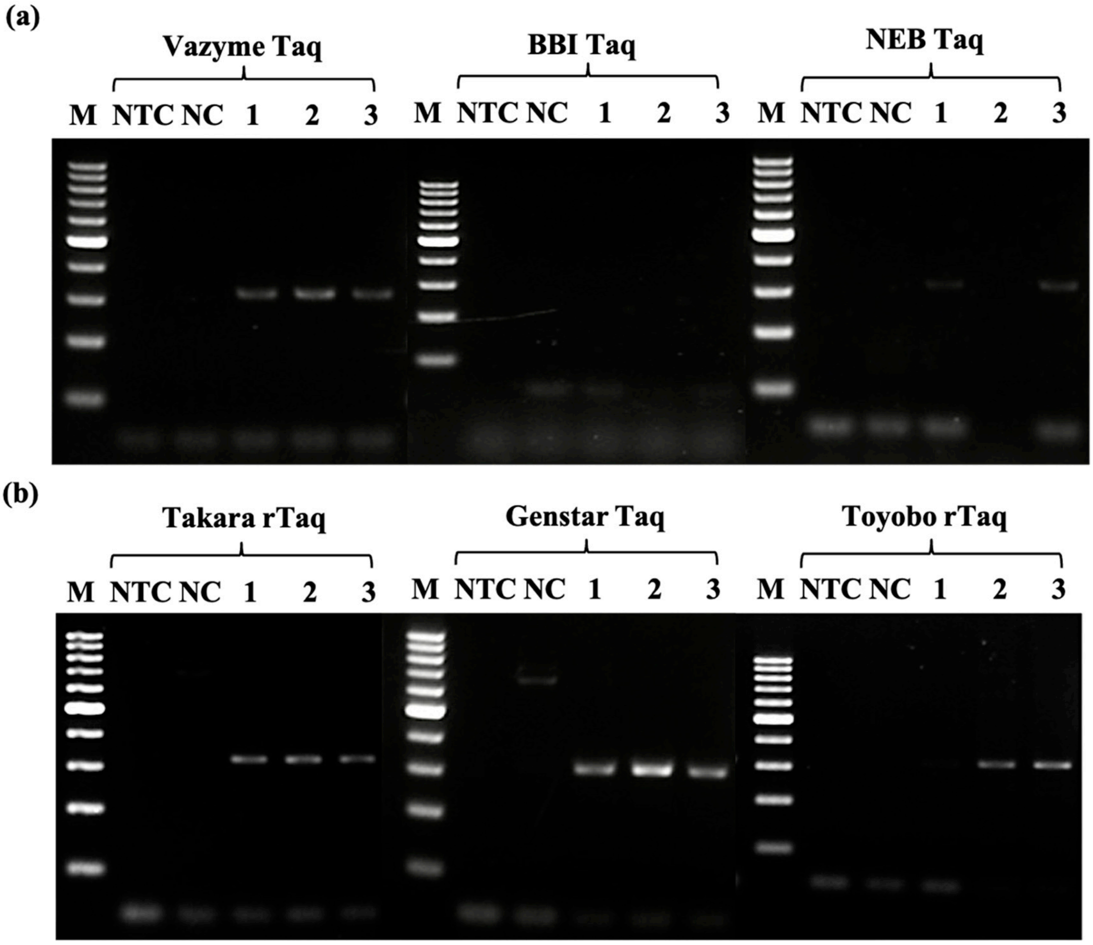

Figure S6. Applicability of using different brands of Taq DNA polymerase in a PCR system with bismuth compound enhanced amplification of the APOE gene. (a) Vazyme Taq, BBI Taq, NEB Taq and (b) Takara rTaq, Genstar Taq, Toyobo rTaq DNA polymerases amplified under the action of ammonium bismuth citrate or bismuth subcarbonate, respectively. Lanes 1-3: 0μM material (3%DMSO and 5%glycerol), 0.022mM ammonium bismuth citrate and 0.02mM bismuth subcarbonate. Lane M: 100bp DNA Ladder (DL1002, Generay); lane NTC: no template control; Lane NC: negative control with template.

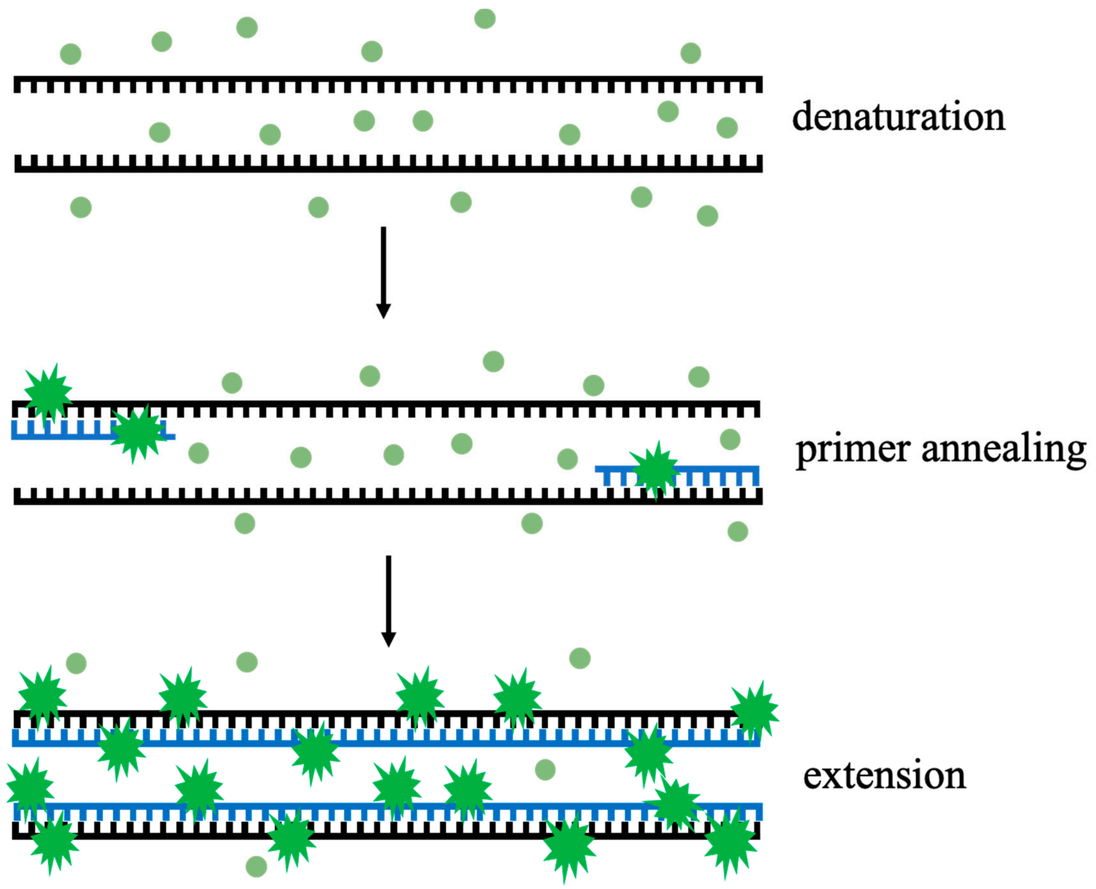

Figure S7. The formation principle of melting curve.
